# A randomized blinded controlled trial of mobile phone reminders on the follow-up medical care of HIV-exposed and HIV-infected children in Cameroon: study protocol (MORE CARE)

**DOI:** 10.1186/1745-6215-14-313

**Published:** 2013-09-25

**Authors:** Jean Joel R Bigna, Charles Kouanfack, Jean Jacques N Noubiap, Claudia S Plottel, Sinata Koulla-Shiro

**Affiliations:** 1 Faculty of Medicine and Biomedical Sciences, University of Yaoundé 1, P.O. Box: 1364, Yaoundé, Cameroon; 2 Faculty of Medicine, University of Montpellier 1, 2 rue Ecole de Médecine, 34060 Montpellier Cedex 2, Montpellier, CS 5001, France; 3 Goulfey District Hospital, Goulfey, Cameroon; 4 Accredited Treatment Centre, Yaoundé Central Hospital, P.O. Box: 5555, Yaoundé, Cameroon; 5 Internal Medicine Unit, Edea Regional Hospital, P.O. Box: 100, Edea, Cameroon; 6 Department of Medicine, New York University Langone Medical Center, 550 First Avenue, New York, NY 10016, USA

**Keywords:** MORE CARE, HIV, AIDS, Children, Attendance, Telemedicine, Short message service (SMS), Phone calls, Resource-limited setting

## Abstract

**Background:**

In Cameroon, only two-thirds of children with HIV exposure or infection receive appropriate HIV-directed medical care. Mortality, antiretroviral therapy resistance and suboptimal virological response are strongly related to missed opportunities for treatment, and, more specifically, to skipped scheduled medical appointments. The present trial, MORE CARE (Mobile Reminders for Cameroonian Children Requiring HIV Care) seeks to determine if reminders sent by text message (SMS), phone call, or concomitant SMS and phone calls most increase the presence at medical appointments of HIV-infected or -exposed children (efficacy), and which is the most efficient related to working time and financial cost (efficiency).

**Methods/Design:**

We will carry out a multicenter single-blind, randomized, factorial controlled trial. A randomization list will be electronically generated using random block sizes. Central allocation will be determined by sequentially numbered. A total of 224 subjects will be randomized into four groups (SMS, Call, SMS + Call, and Control) with an allocation ratio of 1:1:1:1. SMS and calls will be sent between 48 and 72 hours before the scheduled appointment. A medical assistant will send out text messages and will call participants. Our primary outcome is appointment measured by efficacy and efficiency of interventions. We hypothesize that two reminders (concomitant use of SMS and phone calls) as an appointment reminder is more effective to improve appointment compared to one reminder (only SMS or only call), and that the most efficient is use of only SMS. The analysis will be intention to treat.

**Discussion:**

This trial investigates the potential of SMS and phone calls as motivational reminders to improve children’s adherence to medical appointments for HIV-related care in Cameroon. The intervention will act to end missed appointment due to forgetfulness.

**Trial registration:**

Pan African Clinical Trials Registry: PACTR201304000528276

## Background

In Central Africa, vertical transmission of human immunodeficiency virus (HIV) infection from mother to child approaches 30%, reflecting in part inadequate implementation of postnatal preventive strategies, especially during breastfeeding [1]. In Cameroon, the number of new pediatric HIV infections was increasing and reached 6,800 in 2011 [2]. Recent estimates indicate that only 13% of children requiring antiretroviral HIV therapy (ART) actually receive it [2]. Inadequate attendance at medical follow-up visits in infancy is a potentially reversible factor. Only 65% of infants born to seropositive mothers presented to the recommended six-weeks-of-age medical visit [3]. Many factors underlie nonattendance at scheduled visits for HIV care and loss to medical follow-up. These include intrapersonal factors such as the cost of transportation, food availability, time constraints due to concomitant work, fear of disclosure of HIV status for mother and child, parental perception that the child is healthy, and personal religious beliefs. Interpersonal factors like male partner nonparticipation, familial stigmatization and conflicts play a role as well. Community factors include accepted cultural norms, changing community dynamics, and perceived stigma; these factors, along with health care system factors such as the clinic location, lack of patient-centered care, delays at the clinic, and different appointment scheduling for mother and child contribute to non-adherence with medical visits [4].

Bastard *et al*. have defined an adherence indicator based on timeliness of clinic attendance [5]. The indicator is strongly predictive of virological response to ART and of the occurrence of drug resistance and identifies non-adherent patients in a timely manner in settings where viral load monitoring is not available [5]. Mortality in HIV-infected infants has significantly decreased in the era of effective ART, yet infrequent or sporadic clinic attendance and poor compliance with cotrimoxazole prophylaxis and/or ART in infants born to HIV-infected women and late presentation of infants identified after birth are major contributors to persistent childhood mortality [6]. Adherence with medical follow-up appointments is crucial for optimal treatment of chronic diseases, including pediatric HIV. The World Health Organization (WHO) encourages the use of new technologies to assist health delivery in resource-limited settings [7]. The use of mobile phone text message (SMS) and voice phone calls to enhance attendance at medical appointments shows a favorable effect on clinic attendance in the general population as well as among adults with HIV/acquired immunodeficiency syndrome (AIDS) [8]. There is also evidence from randomized controlled trials that mobile phone text-messaging at weekly intervals is efficacious in enhancing adherence to ART and in improving HIV viral load suppression [9]. Some studies on attendance of medical appointment have been conducted among adults and adolescents [10-17]. A recent Cochrane review concludes that appointment reminders sent via mobile phone text messaging (SMS) are as efficacious as voice phone call reminders in increasing patient attendance at medical visits and the effect is greater for both SMS and phone calls interventions as compared to no reminder [17]. These findings are similar to those of Hasvold *et al*. [18]. To our knowledge, the effect in developing countries of medical appointment reminders delivered by SMS, by mobile phone call, or by both SMS and phone call on clinic attendance of babies and children requiring HIV-related care has not yet been studied. The proposed trial, **mo**bile **re**minders for **Ca**meroonian children **re**quiring (MORE CARE) HIV treatment, aims to determine the reminder method that most increases pediatric patient attendance at medical appointments for treatment of HIV-infected and HIV-exposed children, and to determine which is the most efficient related to working time and financial cost.

## Methods/Design

The trial is registered with the Pan-African Clinical Trials (http:pactr.org) as PACTR201304000528276.

### Trial design

**Figure 1:**
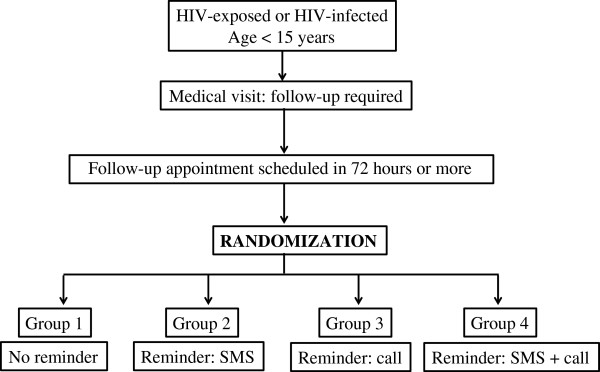
**Schematic of MORE CARE study groups.** We propose to enroll 224 adult-child participant pairs who will be randomized equally into four groups, as described in the text.

**Figure 2:**
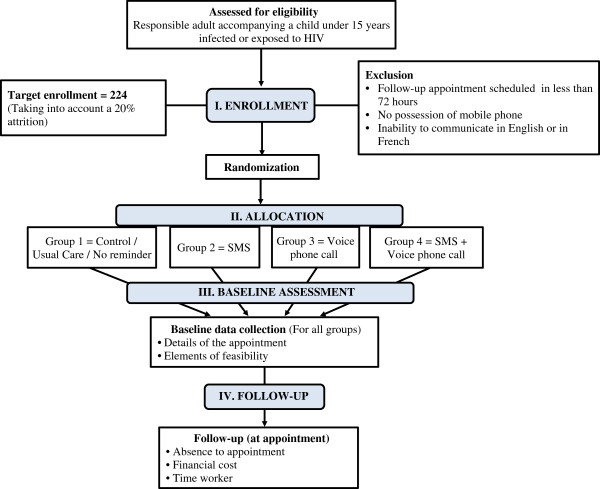
**Diagram of the study according to the recommendations of the Consolidated Standards of Reporting Trials (CONSORT) [**[19]**].**

We will carry out a 2 x 2 factorial, multicenter randomized controlled trial, single-blind with an allocation ratio of 1:1:1:1 for adult-child pairs to four groups. Group 1 will serve as the control group and is composed of subjects provided an appointment and no reminder, reflecting the usual standard of care. Group 2 subjects will be provided an appointment and an SMS reminder. Group 3 members will be provided an appointment and a voice phone call reminder. Group 4 members will be provided both an SMS and a voice phone call reminder. A schematic of the groups and a study diagram according to the recommendations of the Consolidated Standards of Reporting Trials (CONSORT) [19] are shown in Figures 1 and 2 respectively. The study will be performed for 5 months, from January to May 2013.

### Randomization

Randomization assignments will be generated in blocks of four for each study site and will be allocated electronically using WinPepi version 11.25 [20]. After data collection of eligible subjects (adult-child pairs) who have given their consent, we will randomly assign participants into four groups. Allocation and randomization will be made centrally. In each block that is randomly pre-constituted, each subject (adult-child pair) will be allocated to one group.

### Blinding

The treating physician, the medical administrative assistant who will contact participants in Groups 2, 3, and 4 via mobile phone, the nurse (outcome assessor) responsible for recording patient presence or absence at the appointment, and analysts will be blinded. The participant will be received at the reception, and after giving his/her written consent, data will be collected by a nurse. The nurse will then send the following information to the medical administrative assistant: the identification code of the participant, date of appointment, the physician’s name, the phone number of the participant and the language of communication (French or English). A medical administrative assistant will then be responsible for carrying out all intervention: the sending of SMS and the calls to participants. The appointment will then be notified by the nurse of the reception. Before data analysis, we will assign a code for the four groups.

### Demographics and study sites

Cameroon is a sub-Saharan African country. In 2010, there were 19,406,100 people distributed in 10 regions [3]. Data from routine antenatal screening indicates a HIV infection rate of 8.4% in 2011 among pregnant women. Furthermore, early diagnosis in infants born to HIV positive mothers showed a positivity rate of 7.1% [3]. The MORE CARE study research sites are located in two regions of Cameroon. The Essos National Insurance Fund Hospital is an urban setting located in Yaoundé, the administrative headquarter of the Centre Region and the political capital of Cameroon. It is situated in the Centre Region. The prevalence of HIV in Yaoundé is estimated at 6.4% in the population aged between 15 and 49 years old [21]. The Kousséri Regional Hospital and Goulfey District Hospital, which are respectively a semi-urban and a rural setting, are located in the Far North Region of Cameroon. This region has the lowest prevalence of HIV infection in Cameroon (1.2% in the population aged 15 to 49 years) [21]. The rate of loss to follow-up at sixth week after birth of children born to HIV infected mothers is 17.0% in the Centre region and 13.3% in the Far-North region, with a national prevalence of 35.1%. By twelve months of age, only 66% of these children are seen for serological diagnosis of HIV infection [3].

### Participants

We will include in the study all persons aged 18 years or older accompanying a HIV-infected or exposed child aged less than 15 years for HIV care and consenting to participate in the study. We will exclude to randomization i) subjects who do not have a mobile phone; ii) subjects whose follow-up appointment is scheduled in less than 3 days; and iii) subjects who are unable to communicate, read, or write in English or French.

### Reminder intervention

**Figure 3:**
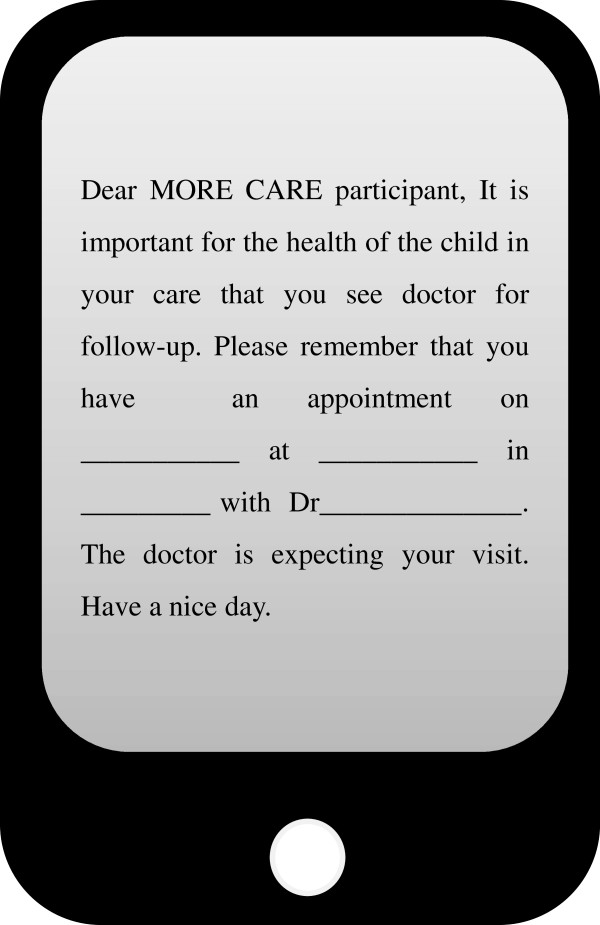
**Text message.** We will send this text message to all participants who allocate to Group 2 (text message (SMS)) and Group 4 (SMS + call).

In the group receiving both SMS and phone calls (Group 4), the SMS reminder, in French or in English, will be sent between 08:00 and 12:00 AM, 72 hours before the appointment followed by a reminder phone call 48 hours before the appointment. The SMS will be sent manually. The SMS will serve as an appointment reminder and motivator. It will contain the date and the time of appointment, the location, and the name of the treating physician (Figure 3). To comply with medical confidentiality, the message will not contain any information on the health status of the child. Each text message sent will include about 300 characters including spaces. The method ‘message received’ will be used to ensure that the message was actually sent. For calls, a medical administrative assistant will phone 48 hours before the scheduled appointment, between 08:00 and 12:00 AM with communication in French or in English. A maximum of three calls attempts will be made. During the phone call, the medical secretary will remind the participant of the date and the location of his/her appointment and of the name of the treating physician. The name of the participant or the child will not be mentioned. In both SMSs and phone calls, communication is unidirectional towards the participant who cannot in return contact the medical secretary. This phone call procedure will be identical for the group receiving only phone calls (Group 3). Concerning the group receiving only the SMS (Group 2), the same procedure for sending SMS will be followed, and the SMS will be sent 48 hours before the appointment instead of 72 hours. It is important to note that, the content of communication (by SMS or by voice call) will be the same for all interventions.

### Control

The control group (Group 1) will receive neither SMS nor phone calls, reflecting the usual practice of care.

### Study objectives

The objective of this trial is to determine which reminder - SMS, phone call, or both SMS and phone calls - is the optimal method to increase HIV-infected and HIV-exposed children’s attendance at medical appointments (effectiveness) and which is the most efficient related to working time and financial cost (efficiency).

### Outcome measures

#### Primary outcomes

Table 1 shows an overview of the outcomes and statistical method that will be used. The primary outcome will be the presence at appointment. It will be measured by effectiveness, and efficiency related to both financial cost and working time. Effectiveness will be the capability of producing a presence at an appointment (attendance). We will measure three aspects of efficiency using the following ratios: i) presence at appointment/financial cost x working time; ii) presence at appointment/financial cost; and iii) presence at appointment/working time. Time for text message intervention will be measured from the start of writing the message until the sending. For phone calls, this time measure will begin when we make the first call (not when the participant picks up the phone) and will stop at the end of the communication. The cost of each call and SMS will be evaluated using a prepaid plan. The prepaid plan means that the phone will have a financial amount of communication known before the call. After call, the difference will be made with the remaining amounts. Each SMS will cost 50 or 100 Francs of Financial Community of Africa (FCFA) (approximately 0.076 € or 0.152 €).

**Table 1:** Overview of outcome measures

**Outcomes measures**	**Scale**	**Type**	**Measure**	**Analysis method**
**Primary**				
Presence at appointment				
• Efficacy	Nominal	Binary	% present at appointment	Chi-square test
				Additive and multiplicative scales for synergy measurement
• Efficiency	Ratio	Continuous	Mean of efficiency	*T*-test
- Working time	Ratio	Continuous	Mean of working time	*T*-test
- Financial cost	Ratio	Continuous	Mean of financial cost	*T*-test
**Secondary**				
Success of intervention	Nominal	Binary	% success of intervention	Chi-square test

#### Secondary outcomes

The intervention will be considered unsuccessful if we do not receive ‘message received’ after sending of SMS, or if we call in vain after three attempts between 8:00 and 12:00 am. For those who will receive both SMS and phone call, the intervention will be considered unsuccessful if these two conditions are fulfilled.

#### Sample size

The primary objective is to measure the patient presence at their scheduled appointment. The calculation of the sample size will be based on the comparison of proportions of presence at appointment. The value of alpha is 0.05. The test is two-tailed. We assume that the presence at appointments using SMS as reminder and phone calls is, respectively, 59.0% and 59.6% [11]. We also assume that the difference would be 25% between the group that will receive both SMS and phone calls and the groups that will receive only an SMS or only phone calls. The minimum size of the sample required will be 50 subjects in each group for analysis by intention to treat with 80% power and a significance level of 0.05. We will exclude from analysis the participants with incomplete data. For this we assume a 10% attrition rate. Thus, we will need 224 subjects (56 in each group). WinPepi has been used to calculate the size of the sample [20].

### Analysis plan

Data will be coded, entered, and analyzed using the Statistical Package for Social Science (SPSS) version 20.0 for Windows (SPSS, Chicago, Illinois, USA). We will describe continuous variables using means with standard deviations, and categorical variables using their frequencies and percentages. We will use the principle of ‘intention to treat’ analysis. The risk ratio will be used to measure effect of interventions on proportion of presence at medicals appointments and will be expressed with 95% confidence interval. The *t*-test will be used to compare quantitative variables, and the chi-square test will be used to compare qualitative variables. A *P* value less than 0.05 will be considered statistically significant. WinPepi will be used to measure the interaction between SMS and call in the group receiving both call back methods of appointment.

### Additional studies

We will measure in MORE CARE study, the difficulties to use mobile phone communications as appointment reminder tool in sub-Saharan Africa. These obstacles include:

1) Non possession of mobile phone: Possession of mobile phone will be evaluated by a yes/no question. The proportion of patients with mobiles will be compared between the three settings of the study (urban, semi-urban, and rural).

2) Obstacles on the methods and language of communication: The written communication (communication by text message) will be evaluated and will be accepted as satisfactory if the participant reads and understands the consent form. Oral communication (communication by phone call) will be accepted as satisfactory if the participant understands the questions ‘What is your name?’, ‘How old are you?’, and ‘Where do you live?’. We will compare ability to use oral and written communication. We will also compare types of communication between the three settings of the study.

3) Refusal to participate: We will measure the proportion of subjects who refuse to receive SMS or phone calls. We will compare this refusal between settings and between SMS and calls.

### Ethical considerations

The trial was approved by the Faculty of Medicine and Biomedical Sciences, University of Yaoundé 1. The trial was approved by National Research Ethics Committee for Human Health of Cameroon (Ethical approval N° 2013/03/232/L/CNERSH/SP). The study will be conducted in accordance with Declaration of Helsinki [22]. Prior to enrollment, all eligible participants will provide written informed consent.

## Discussion

Some studies investigating the use of mobile text message and phone calls to improve adherence to medicals appointments showed various results [10-13,15-18]. Unlike those studies, our MORE CARE trial will measure a possible synergic interaction of combining text message and phone call. Globally, the use of SMS and phone call reminders in adult studies improve appointment attendance. Improving adherence to scheduled medical visits can play a key role in better management of chronic diseases in resource limited setting. Findings of this trial can be generalized to other chronic diseases. The MORE CARE trial therefore offers a significant an opportunity to promote health in low-income countries using existing mobile cellular technology.

The choice of a multicenter study involving three regions different in their socioeconomic and demographic characteristics, and urban, semi-urban and rural settings will allow us to have a better estimation of the difficulties in using mobile phone technology in resource limited setting; this will provide supplementary information and complete the findings of Mbuagbaw *et al*. who conducted a singled-center randomized controlled trial on mobile phone text messaging versus usual care for improving adherence to highly active ART in Cameroon [23].

A major potential risk of use of mobile phone would be an accidental disclosure of HIV status of children. This potential effect will be explained clearly to the participant. This is why we choose not to mention the status of children during call and in text messages. In similar trial in Uganda, there was no issue of privacy and confidentiality [14]. In a trial in Cameroon, using text message for improving adherence to ART, one female withdrew from the study because she felt it had compromised her confidentiality [23]. In developing countries, fear of non-respect of personal data privacy can also limit the use of text message reminders [24].

Compared to SMS, which is not interactive, a phone call is a better channel for message delivery to patients or their close relatives. On the other hand, unlike a phone call, in using SMS, if the recipient is not immediately reachable when the message is sent, he/she can read the text message afterwards. In addition, in Africa, some barriers like timing of messages, mobile network fluctuations, and mobile phone turnover can reduce the potential use of mobile phone technology to improve adherence to treatment [24]. Other barriers include language impediments and lack of financial incentive.

Some factors associated with non-adherence to ART in the adult population in Cameroon can influence presence at appointment: high monthly income, binge drinking, drug and tobacco use, lack of family support for adherence, potential for experiencing discrimination and stigma, switching regimen, increased duration on medication, cost of care, increased distance from clinic, large hospital size, and no task shifting from physician to other staff [25]. In some previous studies, the sending of SMS was the less financial cost method compared to voice phone call [17]. The use of mobile phone reminder in this trial can reduce impact of some correlates of nonattendance to medical appointments in Cameroon.

Finally, the study will possibly contribute to increasing evidence of the usefulness of mobile phone technology for promoting health in low- to middle-income countries like Cameroon [26].

### Trial status

The recruitment is ongoing.

## Abbreviations

ART: Anti-retroviral therapy; CONSORT: Consolidated Standards for Reporting Trials; FCFA: Francs of Financial Community of Africa; HIV: Human immunodeficiency virus; MORE CARE: Mobile Reminders for Cameroonian Children Requiring HIV Treatment; PMTCT: Preventing mother-to-child transmission; SMS: Short message service, also text message; WHO: World Health Organization.

## Competing interests

The authors declare that they have no competing interests.

## Authors’ contributions

JJRB conceived the study and drafted the manuscript. CK participated in the study design and assisted in drafting the manuscript. JJNN, CSP and SKS critically revised the manuscript. All authors have given final approval of the version to be published. All authors read and approve the final manuscript.
